# Protease inhibitors effectively block cell-to-cell spread of HIV-1 between T cells

**DOI:** 10.1186/1742-4690-10-161

**Published:** 2013-12-24

**Authors:** Boghuma Kabisen Titanji, Marlen Aasa-Chapman, Deenan Pillay, Clare Jolly

**Affiliations:** 1 Division of Infection and Immunity, University College London, Cruciform Building, Gower St, London WC1E 6BT, United Kingdom; 2 Africa Centre for Health and Population Sciences, University of KwaZulu Natal, KwaZulu-Natal, South Africa

**Keywords:** HIV-1, Virological synapse, Cell-cell spread, Protease inhibitor, ART

## Abstract

**Background:**

The Human Immunodeficiency Virus type-1 (HIV-1) spreads by cell-free diffusion and by direct cell-to-cell transfer, the latter being a significantly more efficient mode of transmission. Recently it has been suggested that cell-to-cell spread may permit ongoing virus replication in the presence of antiretroviral therapy (ART) based on studies performed using Reverse Transcriptase Inhibitors (RTIs). Protease Inhibitors (PIs) constitute an important component of ART; however whether this class of inhibitors can suppress cell-to-cell transfer of HIV-1 is unexplored. Here we have evaluated the inhibitory effect of PIs during cell-to-cell spread of HIV-1 between T lymphocytes.

**Results:**

Using quantitative assays in cell line and primary cell systems that directly measure the early steps of HIV-1 infection we find that the PIs Lopinavir and Darunavir are equally potent against both cell-free and cell-to-cell spread of HIV-1. We further show that a protease resistant mutant maintains its resistant phenotype during cell-to-cell spread and is transmitted more efficiently than wild-type virus in the presence of drug. By contrast we find that T cell-T cell spread of HIV-1 is 4–20 fold more resistant to inhibition by the RTIs Nevirapine, Zidovudine and Tenofovir. Notably, varying the ratio of infected and uninfected cells in co-culture impacted on the degree of inhibition, indicating that the relative efficacy of ART is dependent on the multiplicity of infection.

**Conclusions:**

We conclude that if the variable effects of antiviral drugs on cell-to-cell virus dissemination of HIV-1 do indeed impact on viral replication and maintenance of viral reservoirs this is likely to be influenced by the antiviral drug class, since PIs appear particularly effective against both modes of HIV-1 spread.

## Background

Combination antiretroviral therapy (cART) for the treatment of Human Immunodeficiency Virus Type-1 (HIV-1) infection is very effective and has transformed Acquired Immunodeficiency Syndrome (AIDS) from a fatal disease into a manageable chronic condition. Despite the success of existing therapies in controlling viral replication and preventing disease progression, treatment is not curative and remains a life-long commitment for infected patients. The ability of the virus to persist in reservoirs within the body, re-emerge in the face of therapeutic lapses and to evolve drug resistant variants continues to frustrate the efforts towards finding a definitive cure. A good understanding of the mechanisms of viral persistence in the context of antiretroviral therapy is crucial for developing novel eradication strategies.

Cellular reservoirs are recognized key drivers of viral persistence within the host [1]. Ongoing viral replication in patients receiving cART is debated as a mechanism for viral persistence with opposing lines of evidence both in support of [2-4] and against [5-7] this mechanism of persistence. In support of full suppression of viral replication with cART, patients with good adherence to treatment do not show evidence of viral evolution and treatment failure; also no further decrease of residual viremia is seen with intensification of cART regimens [5-7]. However, recent treatment intensification studies with the Integrase Inhibitor (INI) Raltegravir have noted an increase in episomal DNA and a reduction in the size of the latent reservoir [2-4]. These studies suggest that there is ongoing residual viral replication, which may be suppressed by addition of INIs to existing treatment regimens.

HIV-1 can disseminate between CD4+ T lymphocytes either by cell-free infection or by cell-to-cell spread at virological synapses (VS). Cell-to-cell spread is a very efficient mode of retroviral dissemination, which allows for directed virus transfer across a virological synapse, obviating the need for prolonged cell-free virus diffusion [8-10]. Notably, this mode of virus spread is several orders of magnitude more efficient than classical cell-free diffusion [11-13] and may be more resistant to neutralization by antibodies [10,14-17]. Furthermore, it has been proposed that the replicative advantages conferred by cell-to-cell spread, mediated during physical contact between infected and uninfected cells, may be important in lymphoid tissues where CD4+ T lymphocytes are densely-packed and likely to frequently interact. Indeed, studies using intravital imaging have validated the concept of the VS *in vivo*[18,19]. More recently it has been suggested that cell-to-cell virus transfer may be a mechanism by which HIV-1 can evade ART, and thus continue to replicate at low levels in the presence of ongoing therapy [20]. In that study, Sigal *et al.* proposed that the large number of viral particles which are transmitted to an uninfected target cell during cell-to-cell transfer increases the probability that at least one viral particle will stochastically escape inhibition by drugs and proceed to infect the cell [20]. They tested this hypothesis by assessing the effects of RTIs on virus spread in an *in vitro* experimental model and showed that cell-to-cell spread was less sensitive to inhibition by RTIs than cell-free transmission [20]. A similar mechanism of saturation of inhibitors by a large pool of incoming virus particles has also been suggested to explain the resistance of cell-to-cell virus transfer to inhibition by innate, antiviral cellular factors [21,22]. However, in a conflicting report Permanyer *et al.* conducted similar *in vitro* assays and reported that RTIs were equally effective at blocking both modes of HIV-1 dissemination [23]. The disparity in these studies therefore raises questions regarding the true impact of antiretrovirals on cell-to-cell HIV-1 transmission. Moreover, because both studies restricted their analysis to RTIs it remains unclear whether the different drug classes that constitute cART vary in their ability to block cell-to-cell spread of HIV-1.

Protease Inhibitors constitute an important component of cART by virtue of their potency and the high barrier that they impose against selection of drug resistant variants [24,25]. PIs are the only class of antiretroviral drugs, which have been tested for use as monotherapy for the treatment of HIV and shown to be not inferior to cART regimens in maintaining suppression of viral replication [26,27]. While PIs are mostly reserved for use in 2nd line therapy in developing countries when 1st line therapies fail, the rise in circulating baseline resistance to RTIs in treatment naïve individuals [28,29] has led to increased use of PI-based cART for first-line treatment, making this drug class particularly important for the future of HAART. PIs are known to act by preventing cleavage of viral polyproteins into functional subunits, thereby inhibiting maturation of the virus. A recent study has suggested that in mediating their antiviral effects, PIs affect multiple distinct steps in the life-cycle of the virus including both entry and post-entry events explaining their remarkable potency in suppressing viral replication [30]. During cell-to-cell spread, virus assembly and budding are polarized towards the cell-cell interface [9,10]. Therefore it is possible that *de novo* viral HIV-1 assembly and maturation at the VS, coupled with more rapid virus transfer, might limit the efficacy of PIs during cell-to-cell spread. However the impact of PIs on cell-to-cell transfer of HIV-1 has not been investigated.

Here we have specifically compared the relative efficacy of PIs during cell-free and cell-to-cell spread of HIV-1 between T lymphocytes. We find that PIs (Lopinavir and Darunavir) are equally effective at blocking both modes of HIV-1 spread at similar IC50 concentrations. We also show that a mutant of HIV-1 containing well-defined Lopinavir resistance mutations retains its resistance profile during cell-cell spread. By contrast we observe that cell-to-cell spread of HIV-1 is less inhibited by RTIs but note intra-class differences in the ability of RTIs to block cell-to-cell spread, with some NRTIs being far less effective than NNRTIs. Taken together these data reveal that while PIs are potent inhibitors of cell-to-cell spread, different classes of antiretroviral drugs display variable efficacy against different modes of HIV-1 dissemination. Thus if cell-to-cell spread of HIV-1 does indeed impact ongoing viral replication and maintenance of reservoirs in treated patients, it does so in a drug-class dependent manner.

## Results

### Protease inhibitors effectively inhibit cell-to-cell transfer of HIV-1

**Figure 1:**
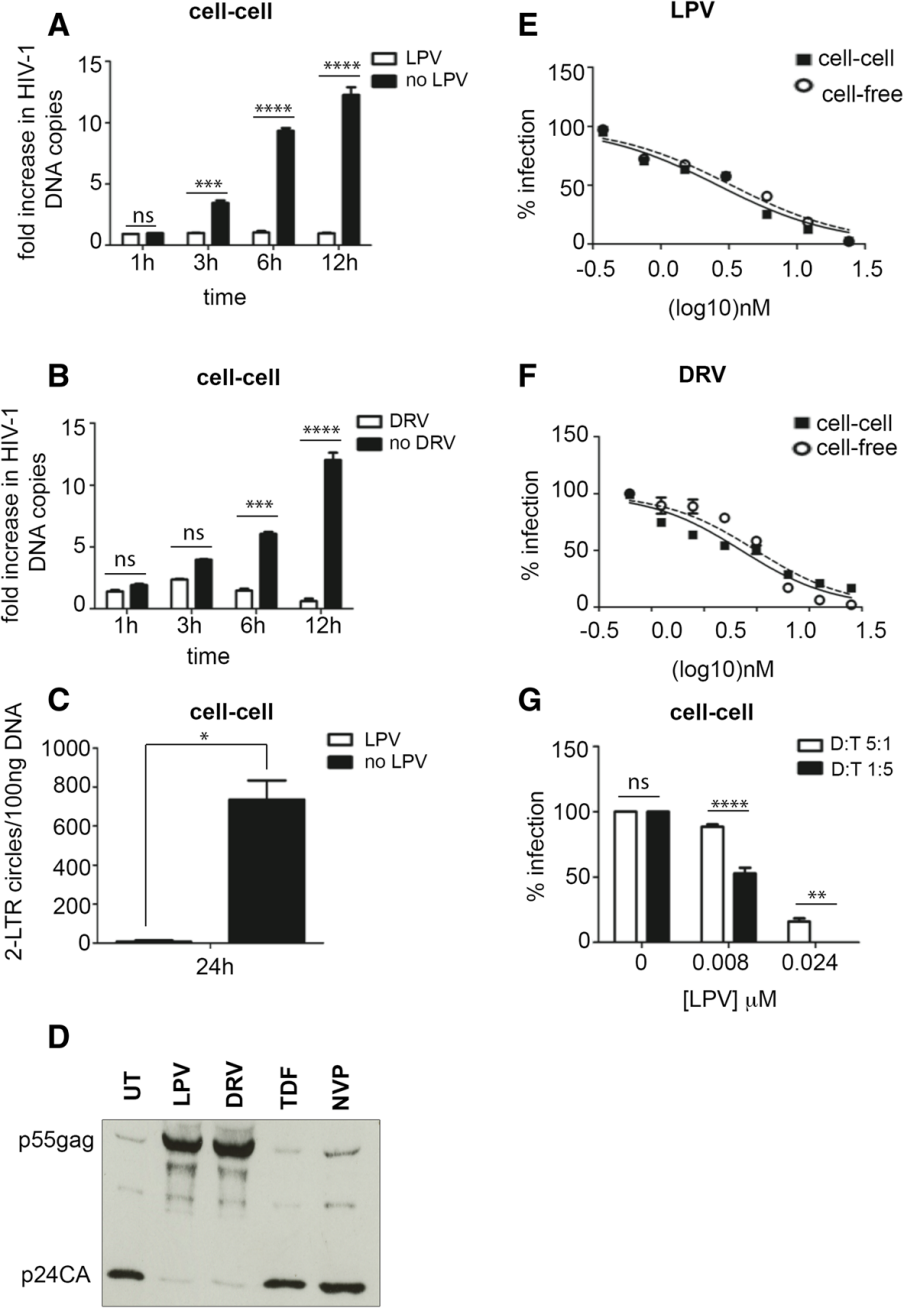
**Protease Inhibitors effectively block cell-to-cell spread of HIV-1. A)** Quantification of cell-to-cell spread of HIV-1 over time in presence of C_max_ LPV (14 μM) and **B)** C_max_ of DRV (12 μM). HIV-1infected Jurkat cells (donors) incubated with PI for 24 h and mixed with uninfected Jurkat cells (targets) with or without PIs. After DNA extraction, qPCR performed to measure appearance of HIV *pol* DNA. Data normalized to *albumin* housekeeping gene and expressed as the fold-increase in HIV DNA copy number over time relative to the baseline value at t = 0 h. Data show mean of triplicates, error bars represent the standard deviation of the mean (SD), **** P < 0.0001, ***P<0.001, ns: not significant, two-way ANOVA plus Bonferroni post-test. **C)** Reduced detection of 2-LTR circles following cell-to-cell spread of HIV-1 in the presence of LPV (14 μM). After 24 h co-culture of donor cells and target cells with or without LPV, 2-LTR circles were detected by qPCR. ** P < 0.05, Unpaired Student T-test. **D)** Confirmation of PI Gag maturation defect in HIV-1. HIV-1+ donor cells incubated with PIs, RTIs or left untreated for 24 h. Virus-containing supernatants harvested, purified and equal volumes of virus analyzed by SDS-PAGE and western blotting for HIV-1 Gag. **E)** LPV and **F)** DRV similarly inhibit cell-to-cell and cell-free HIV-1 spread. HIV-1 infected cells incubated with serial dilutions of PI for 24 h, mixed with target cells and HIV-1 DNA measured as described for **A.** For cell-free infections, virus-containing supernatant harvested from infected donor cells incubated with PI for 24 h was used to infect target cells. 24 h post-infection, DNA was extracted from pelleted target cells and qPCR performed. Error bars represent the SD of the mean of triplicates **G)** Increasing donor: target cell ratio in co-culture reduces the efficacy of LPV in blocking cell-to-cell spread of HIV-1. ****P < 0.0001, ***P < 0.001, ns: not significant, Two-way ANOVA with Bonferroni post-test.

To investigate the effect of PIs on cell-to-cell spread of HIV-1 we used a well-established T cell co-culture system, the validation of which is extensively described elsewhere [31-34]. HIV-1 infected Jurkat T cells were either untreated or pre-incubated with the PI Lopinavair (LPV) or Darunavir (DRV), incubated with uninfected target T cells (Jurkat-1G5) and cell-to-cell spread of HIV-1 was measured by quantitative real-time PCR (qPCR) to enumerate the appearance of *de novo* HIV-1 DNA *pol* copies that arise from reverse transcription within the newly infected T cell population. It has previously been confirmed that using a synchronous population of HIV-1 infected cells (>90% of Gag positive by flow cytometry) this assay reliably measures virus infection of target cells mediated by cell-to-cell spread with little or no contribution from cell-free virus transfer that is significantly less efficient [32,33]. Treated and untreated infected cells used in our assay had comparable Gag positivity after staining and analysis by FACS with mean fluorescence intensity of 109 and 103 for treated and untreated donor cells respectively. Figure 1 shows that (as expected) we observe a time-dependent increase in the appearance of HIV-1 *pol* DNA indicative of cell-cell spread within the control sample that was incubated in the absence of PI (Figure 1A and B). Notably, cell-to-cell spread of HIV-1 was potently blocked in the presence of both Lopinavir and Darunavir at doses corresponding to the maximum plasma concentrations (C_max_) (14 μM LPV; 12 μM DRV) achieved *in vivo*[35-37], with no increase in HIV-1 DNA detected during co-culture in the presence of drug (Figure 1A and B). Inhibiting *de novo* synthesis of reverse transcripts by blocking cell-to-cell spread would also be expected to impact on the appearance of 2-LTR circles that are used as a marker of HIV-1 nuclear import a step that immediately precedes proviral integration [38]. We found that while 2-LTR circles were readily detected (an average of 635 copies/100 ng of DNA at 24 h) in co-cultures performed in the absence of PI, significantly fewer 2-LTR circles were detected for co-cultures performed in the presence of 14 μM LPV (<50 copies/100 ng of DNA) (Figure 1C). Taken together these data suggest that PI can effectively inhibit HIV-1 cell-to-cell spread. To confirm the appropriate activity of PIs on Gag maturation and to ensure there was no defect in overall Gag budding, we performed western blot analysis of purified virus collected from PI treated HIV-1 infected T cells. Donor cells treated with PIs (C_max_ of LPV and DRV) showed the expected predominance of uncleaved p55Gag protein in virions whereas untreated cells or cells treated with RTIs (C_max_ TDF and NVP) contain predominantly p24CA indicative of appropriate protease-mediated Gag cleavage (Figure 1D).

### Protease inhibitors are equally effective at blocking cell-free and cell-to-cell spread of HIV-1

To determine the efficacy of PIs over a range of concentrations, and to compare inhibition of cell-free and cell-to-cell spread, HIV-1 infected donor cells were co-cultured with target cells in the presence of serial dilutions of LPV and DRV and cell-to-cell spread was quantified by qPCR to calculate the concentration at which 50% infection was inhibited (IC50). To measure cell-free infection, the HIV-1+ donor cells were incubated alone and allowed to produce virus in the presence of a dose titration of the PIs. Culture supernatants containing cell-free virus were subsequently harvested, used to infect target cells and infection was quantified by qPCR as described above. Infection curves were generated to determine the IC50 for PIs in both cell-to-cell and cell-free spread (Figure 1E and F and Table 1). Notably, no significant difference was observed in the IC50 of LPV and DRV for either cell-free (3.0 nM and 2.5 nM respectively) or cell-to-cell infection (2.9 nM and 2.8 nM respectively) indicating that PIs are equally effective at inhibiting both modes of HIV-1 spread.

**Table 1:** Summary IC50s for cell-to-cell and cell-free modes of virus transfer with protease inhibitors and reverse transcriptase inhibitors

	**Protease inhibitors**				**Reverse transcriptase inhibitors**					
	**Lopinavir**		**Darunavir**		**Nevirapine**		**Tenofovir**		**Zidovudine**	
	**Cell-cell**	**Cell free**	**Cell-cell**	**Cell-free**	**Cell-cell**	**Cell -free**	**Cell-cell**	**Cell-free**	**Cell-cell**	**Cell-free**
**Mean IC50**	2.9 nM	3.0 nM	2.8 nM	2.5 nM	359.8 nM	86 nM	>80 μM*	7.5 μM	>80 μM	3.4 μM
**SEM**	0.2	0.2	0.4	0.1	89.5	9.2	UD	0.7	UD	0.3
**p-value**	**0.7**		**0.5**		**0.03**		**UD**		**UD**	

SEM = Standard error of the mean calculated from three independent experiments. Because TDF and AZT do not titrate fully for cell-to-cell spread we were unable to estimate IC50s based on the sigmoidal dose response curves generated with GraphPad Prism software. A two-tailed student *t*-test was applied to compare the IC50 values obtained for cell-to-cell vs. cell-free for LPV, DRV and NVP. The *p*-values obtained are shown in the table. UD = cannot be determined. * = Maximum dose of drug tested.

The reduced efficacy of antiretroviral therapy and neutralizing antibodies during cell-to-cell spread has previously been attributed to the higher multiplicity of virus transfer [15,16,39-42]. Therefore we assessed the effect of varying the multiplicity of infection (MOI) on inhibition of cell-to-cell spread by PIs by increasing the number of donor cells in co-culture to achieve a donor-to-target ratio of 5:1, compared to the 1:5 ratio that was used in our previous experiments. Figure 1G shows that increasing the number of HIV-1 infected cells led to a reduced ability of LPV to inhibit cell-to-cell infection when used at a concentration (8 nM) close to the IC50 (Table 1). Increasing the concentration of LPV three-fold (24 nM) restored the ability of LPV to block cell-to-cell spread at the higher donor-to-target ratio (Figure 1G).

### A protease inhibitor drug resistant variant maintains its resistant phenotype in a cell-to-cell assay system

**Figure 2:**
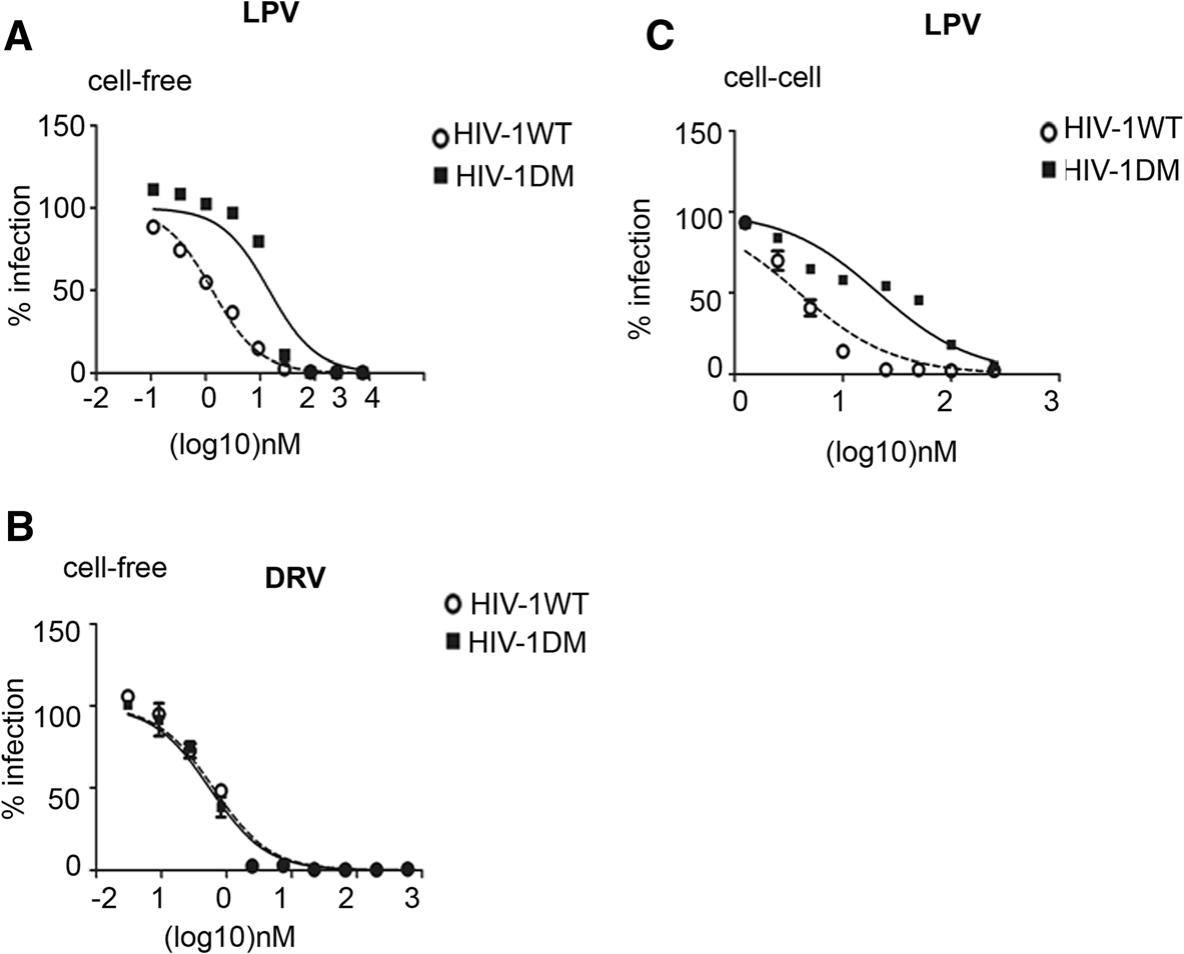
**A PI resistant variant maintains its resistant phenotype during cell-to-cell spread.** HIV-1_DM_ displays enhanced resistance to **A)** LPV but not **B)** DRV when compared to wild-type HIV-1. Cell-free virus was produced from 293T cells in the presence of serially diluted PI, used to infect HeLa TZM-bl cells and infectivity was determined by luciferase assay. Data shown is a representative of two independent experiments. Error bars represent the SD from the mean of duplicates. **C)** HIV-1_DM_ maintains its resistant phenotype in a cell-to-cell spread assay system. Cell-to-cell spread of both resistant and wild-type variants were quantified by qPCR as described in Figure 1 in the presence of a serial dilution of LPV. The dose-infection curves for each virus were fitted with GraphPad Prism software. The data shown are a representative from three independent experiments. The error bars represent the SD of the mean of triplicates.

Next we investigated cell-to-cell spread of a PI drug resistant variant of HIV-1 bearing resistance mutations to LPV and compared this to wild-type virus. This variant has a major drug resistance mutation in Protease (V82A) and a compensatory p7/p1 cleavage site mutation (A431V) in Gag, confers resistance to LPV and is the predominant mutation selected for *in vivo* in patients receiving LPV [43,44]. The PI susceptibility of the mutant virus (termed HIV-1_DM_) was confirmed in a cell-free drug resistance phenotyping assay [45] and was found to be 11-fold more resistant to LPV than the wild-type virus (HIV-1_WT_) (Figure 2A). As expected both viruses were equally susceptible to DRV (Figure 2B). To compare cell-to-cell spread of HIV-1_WT_ and HIV-1_DM_ in the presence of PI, each virus was incubated in the presence of serial dilutions LPV and cell-to-cell spread was quantified by qPCR and IC50s calculated. The drug resistant virus retained its resistance phenotype in a cell-to-cell assay being approximately 7-fold more resistant to LPV than the wild-type virus (IC50 5.6 nM and 37 nM respectively) (Figure 2C). Moreover, the ability of the HIV-1_DM_ to be better transmitted by cell-to-cell spread in the presence of LPV (when compared to wild-type virus) confirms that the inhibition we see in the presence of PI is directly related to the anti-viral drug activity.

### PI can block cell-to-cell spread mediated by HIV-1-infected primary T cells

**Figure 3:**
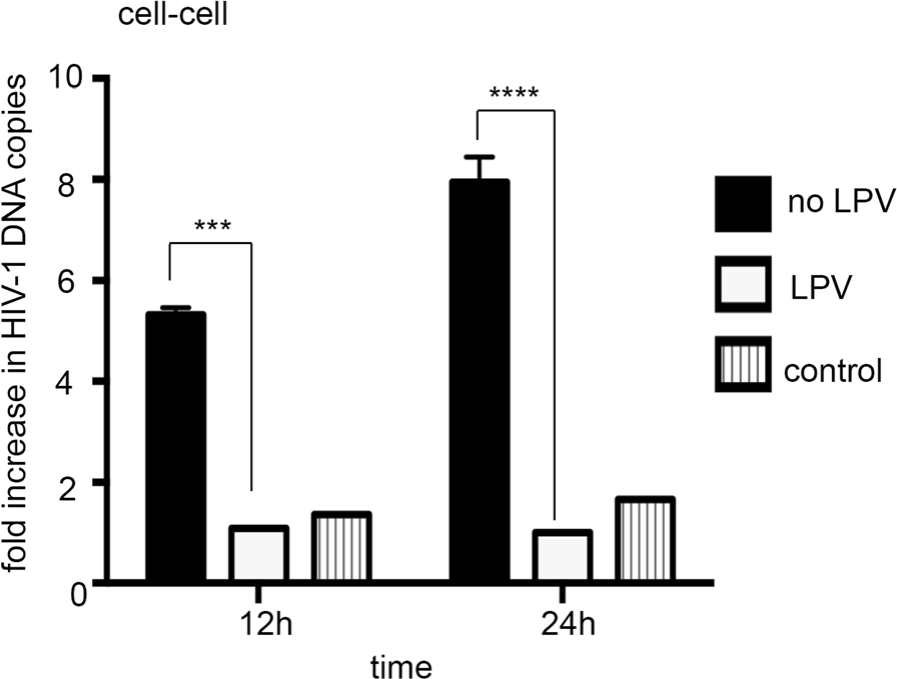
**Protease Inhibitors effectively block cell-to-cell transfer from HIV-1 infected primary T cells: HIV-1 infected primary CD4+ T cells (donor cells) were incubated with LPV (14 μM) for 24 h and mixed with uninfected Jurkat cells (targets) and cell-to-cell spread was quantified by qPCR as described in Figure** 1**.** A representative of two independent experiments performed with two different donors is shown. Data are the mean and error bar represent the SD, **** P < 0.0001, *** P < 0.001, two-way ANOVA with Bonferroni post test.

To determine if PIs were also able to block cell-to-cell spread by HIV-1 infected primary cells, PBMCs were obtained from healthy donors and CD4+ T cells were purified, stimulated with PHA and IL2 and infected with HIV-1. After 72 h these cells were treated with LPV and co-cultured with uninfected target cells and cell-to-cell spread was quantified by qPCR exactly as described in Figure 1. Cell-to-cell spread of HIV-1 mediated by primary CD4+ T cells was blocked by LPV at a dose corresponding to the C_max_ (14 μM) (Figure 3). Because we were unable to achieve >90% infection of primary T cells (60% HIV-1 Gag positive by flow cytometry) an additional control was included in which HIV-1 infected T cells were cultured alone without the addition of fresh uninfected target cells. These data show no increase in HIV-1 *pol* DNA over time indicating that there is no ongoing spreading infection within the primary cell population.

### Reverse transcriptase inhibitors are less effective inhibitors of cell-to-cell spread compared to cell-free infection

**Figure 4:**
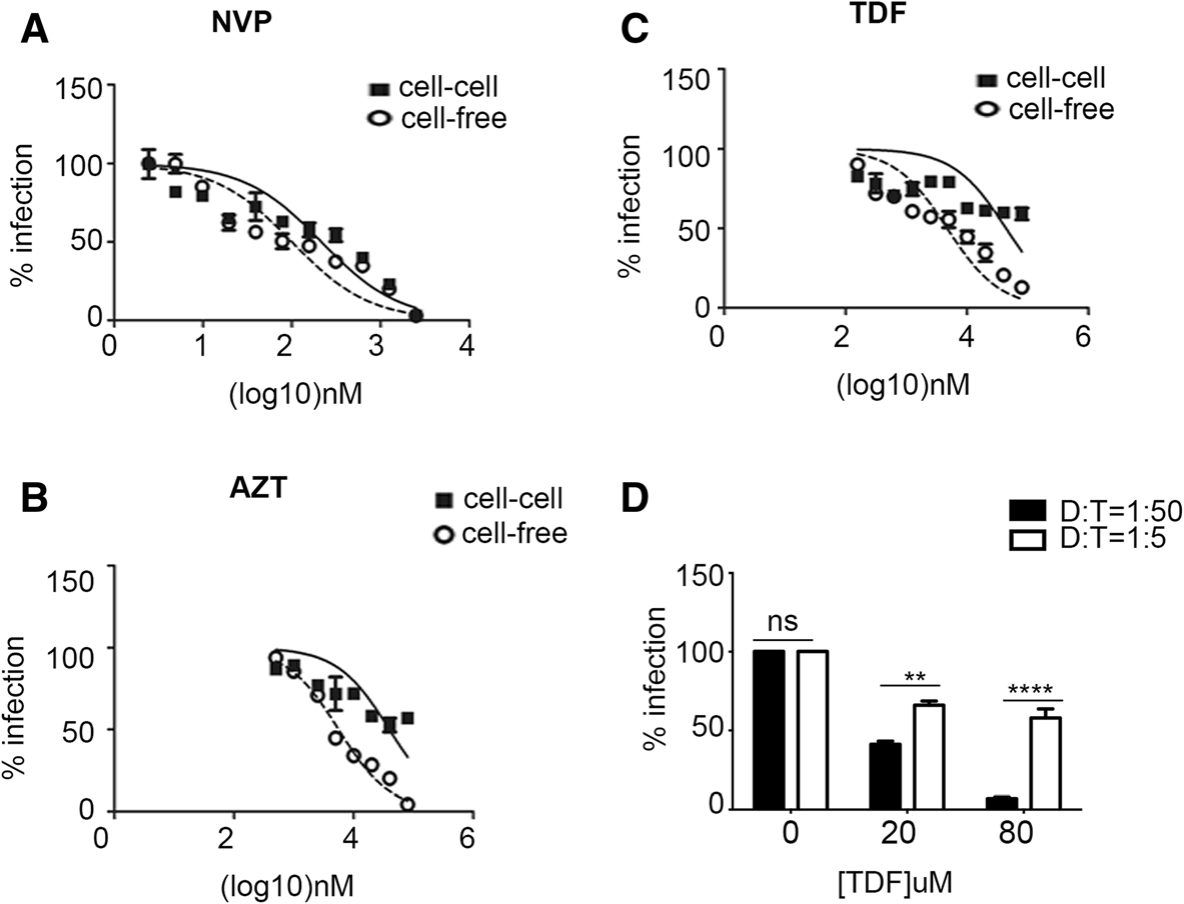
**Reverse transcriptase Inhibitors are less effective inhibitors of HIV-1 cell-to-cell spread compared to cell-free infection.** Uninfected target cells were incubated with serial dilutions of **A)** Nevirapine **(**NVP), **B)** Zidovudine (AZT) or **C)** Tenofovir (TDF) for 1 h and mixed with an equal number of HIV-1 infected donor cells. At 24 h post-mixing, the DNA was extracted and cell-cell spread was measured by qPCR as described in Figure 1. For cell-free infection, virus-containing supernatant harvested from infected donor cells was used to infect target cells in the presence of a serial dilution of the relevant RTI. At 24 h post-infection, the target cells were pelleted, DNA extracted and qPCR was performed. The dose–response curves for both cell-free and cell-to-cell modes of spread were plotted for each drug and curves fitted. Data shown are a representative of three independent experiments. Error bars represent the SD of the mean of triplicates. **D)** Reducing the virus MOI restores the ability of TDF to inhibit cell-to-cell spread. Co-cultures using ten-fold fewer donor cells were performed in the presence of a high, intermediate (IC50) and low dose concentration of TDF. Data show the means of triplicates and error bars represent the SD of the mean, **** P<0.0001, *** P < 0.001, ns: not significant, Two-way ANOVA with Bonferroni post-test.

In light of our results suggesting that PIs are similarly effective at inhibiting cell-to-cell and cell-free spread of HIV-1, we next sought to evaluate the relative efficacy of RTIs in our assay system since conflicting reports exist regarding their ability to block cell-to-cell spread [20,23]. Serial dilutions of Nevirapine (NVP), Tenofovir (TDF) and Zidovudine (AZT) were used in co-culture and cell-free infection assays as described above and the average IC50 values were calculated (Figure 4 and Table 1). Up to four-fold higher concentrations of NVP (p < 0.03) and greater than twenty-fold higher concentrations of TDF were required to achieve a 50% inhibition of cell-to-cell spread compared to cell-free infection (Figure 4A-C and Table 1). Of note, TDF was unable to completely block cell-cell spread even when used at a concentration greater than twenty-fold the IC50 for cell-free transmission. This is in contrast to the data we obtained with PIs, for which a similar concentration of the drugs was sufficient to inhibit both cell-to-cell and cell-free spread (Figure 1D,E and Table 1). Reducing the input of virus-infected cells ten-fold (by decreasing the number of donor cells used in co-culture to achieve a donor-to-target ratio of 1:50) led to an increased ability of TDF to inhibit cell-cell spread (Figure 4D).

### Time of drug addition does not modify the effects of PIs and RTIs on HIV-1 cell-to-cell spread

**Figure 5:**
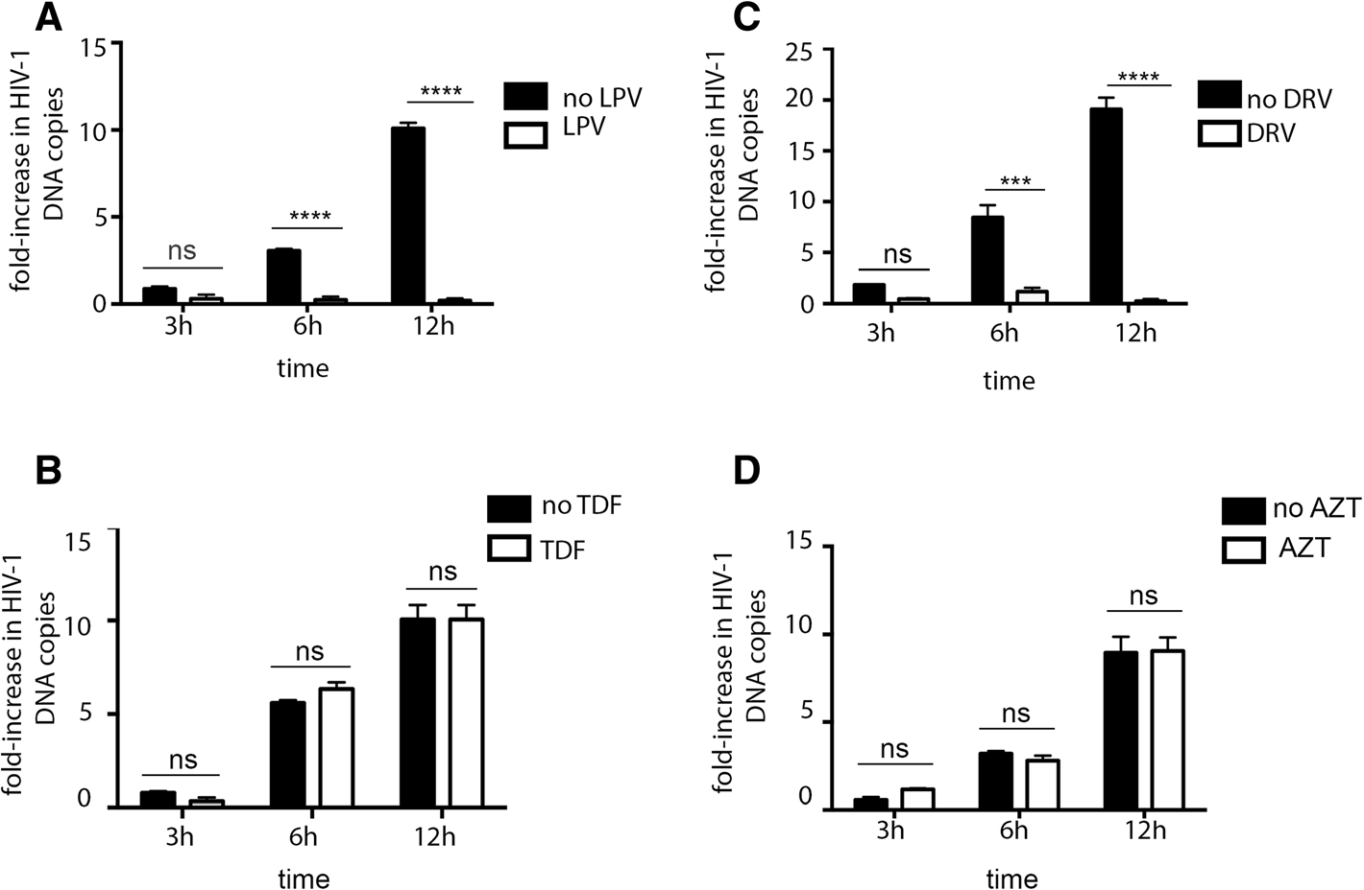
**Time of drug addition does not modify the effects of PIs and RTIs on HIV-1 cell-to-cell spread: HIV-1 infected Jurkat cells (donors) were mixed target cells in the presence of A) of LPV (14 μM) and C) DRV (12 μM) without prior pre-incubation of donor cells with drugs (time of addition t = 0 h).** DNA was extracted at various times post-mixing and qPCR was performed as described in Figure 1. Data show mean of triplicates and error bars represent the standard deviation of the mean (SD). For RTIs, uninfected Jurkat cells (targets) were pre-incubated with **B)** TDF (20 μM) or **D)** AZT (10 μM) for 24 h prior to mixing with infected donor cells. DNA was extracted at various times post-mixing and qPCR was performed. Data show mean of triplicates and error bars represent SD. *** P < 0.001, **** P < 0.0001, ns: not significant, two-way ANOVA with Bonferroni post-test.

We also assessed the effects of time of drug addition on the ability of PIs and RTIs to inhibit cell-to-cell HIV-1 transfer. For PIs, HIV-1 infected donor cells were mixed with uninfected target cells in the presence of LPV (Figure 5A) or DRV (Figure 5C) without prior pre-incubation of donors with the drug (time of addition, t = 0 h). Notably PIs remained effective at inhibiting cell-to-cell virus spread whether drugs were added at t = 0 h (Figure 5) or when HIV-1 infected cells were pre-incubated with drug for 24 h (Figure 1). A similar experiment was performed using RTIs, in which uninfected target cells were pre-incubated with TDF (Figure 5B) or AZT (Figure 5D) for 24 h prior to mixing with HIV-1 infected donor cells and cell-to-cell spread quantified by qPCR. Under these conditions it was found that pre-incubating target cells with the RTIs for 24 h did not improve the ability of TDF and AZT to inhibit HIV-1 cell-to-cell spread. Taken together these data show that time of drug addition does not modify the effects of PIs and RTIs on HIV-1 cell-to-cell spread.

## Discussion

Here we have investigated whether PIs are able inhibit cell-to-cell spread of HIV-1. The results presented herein demonstrate, for the first time, the efficacy of this class of antiretroviral drugs in blocking T cell-to-T cell spread of the virus. By contrast we find that the nucleoside/tide RT inhibitors, Nevirapine, Zidovudine and Tenofovir display reduced efficacy in a cell-to-cell spread model. In particular we note that for Tenofovir, doses up to twenty-fold higher than the maximum plasma concentration (C_max_) achieved *in vivo* did not inhibit cell-to-cell spread more than 50%. These findings may have implications for the efficacy of therapeutic regimens containing NRTIs in contributing to future functional cure approaches, and also within pre-exposure prophylaxis approaches [46-48].

The fact that PIs maintain potency during cell-to-cell spread, whereas RTIs lose activity is likely related to the window of time during which these drugs have to act, their biological function and the multiplicity of infection. PIs prevent cleavage of viral polyproteins into functional sub-units leading to the formation of immature non-infectious virus particles. Exposure of HIV-1 infected cells to a PI would generate a pool of virions unable to mature and result in the transfer of non-infectious virus across the virological synapse. Thus, the production of non-infectious virus would have similar consequences for HIV-1 dissemination by both cell-free and cell-to-cell spread and indeed we find both modes of HIV-1 transmission are inhibited by PIs with similar IC50 values. Cell-to-cell spread of HIV-1 is associated with high multiplicity of infection that is achieved by a combination of polarized virus budding, clustering of virus entry receptors and the close physical contact between cells limiting the requirement for prolonged virus [8,17,33,49,50]. Therefore, RTIs that act within the target T cell may be more easily saturated by the large amount of incoming infectious virions transmitted at virological synapses. In support of this, we found that cell-to-cell spread of HIV-1 was considerably more resistant to RTIs than cell-free infection, in agreement with previous reports [20,42]. The importance of the MOI in this context was further evidenced by showing that altering the relative ratio of infected and uninfected cells impacted on the degree to which PIs and RTIs could inhibit cell-to-cell spread. Interestingly, a recent study suggests that PIs affect multiple distinct steps in the life-cycle of the virus (down-stream of the well-defined block to Gag maturation) including both viral entry and post-entry events [30]. Therefore it is possible that the multiple effects of PIs could also contribute to the particular effectiveness of this drug class at blocking cell-to-cell infection by contrast to RTIs, which affect a well defined single-step in viral replication.

Our results showing that RTIs have sub optimal efficacy in cell-to-cell spread is similar to the observations of Sigal *et al.*[20], who also found that TDF and Efavirenz (EFV) were poor inhibitors of HIV-1 cell-to-cell spread when compared to cell-free virus infection. However, in their assay they found even larger differences between the concentrations of RTI that was required to inhibit cell-to-cell spread compared to cell-free infection [20]. The reasons for the difference in IC50 is unclear but may be influenced by their use of reporter gene and antigen transfer based infection read-outs (which might overestimate the degree of productive infection in target cells following co-culture in the presence of inhibitors) whereas here we have measured viral DNA as a direct readout of infection. By contrast, Permanyer *et al.*[23] found RTIs to be equally effective against both cell-free and cell-to-cell virus transfer. However, that study did not directly compare the IC50 of the inhibitors for both modes of viral infection across a clinically relevant range of drug concentrations as we have done, and this may explain why their assays did not detect the differences in the dose of RTIs required to block cell-to-cell compared to cell-free infection. Another possible source of variation could be that in the study by Permanyer *et al.*, the virus input was adjusted so that cell-free and cell-to-cell spread resulted in a similar percentage of GFP+ infected cells in the untreated condition. Under those conditions, the IC50 of AZT and TDF were found to be equal. However, normalizing the virus input in this way removes the quantitative effects of high-multiplicity infection mediated by cell-cell spread. Here we have used a direct method to measure cell-to-cell virus transfer by detecting *de novo* HIV-1 DNA transcripts by qPCR and have not adjusted virus input to achieve the same levels of infection of target cells, therefore the quantitative effects of cell-to-cell spread will remain. Taking this approach, we find that RTIs are less effective than PIs at blocking cell-cell spread. PI treatment resulted in inhibition of viral DNA synthesis (reverse transcripts), and we infer from these data that the inhibitory effect of PIs on cell-to-cell infection occurs at or before reverse transcription, as expected. To confirm that inhibition of productive infection through cell-to-cell spread by PIs was complete, we also indirectly measured nuclear entry in the presence of PIs by detection of 2-LTR circles in co-cultured cells. Following 24 h of co-culture in the presence of PIs, virtually no 2-LTR circles were detected compared to the no drug control. Although 2-LTR circles are non-functional forms of intracellular HIV-1 DNA they can serve as surrogate markers of nuclear import of viral DNA as well as for the completion of reverse transcription [38]. We were also able to confirm this effect of PIs on cell-to-cell spread of HIV-1 mediated by HIV-1 infected primary CD4+ T cells.

Here we have used a well-validated laboratory isolate of HIV-1 to evaluate the relative efficacy of PI during cell-cell spread of HIV-1. Currently there is no compelling evidence that HIV-1 subtypes need to be considered in the choice of first or second line cART; however it would be informative in future work to expand these studies and include a panel of different HIV1- strains and clinical isolates, to evaluate other drug classes such as integrase inhibitors and consider the effectiveness of combination therapy during cell-free and cell-cell spread. Furthermore, while PI based intensification studies to date have not shown any reduction in the viral load of patients, these studies have measured plasma viremia [51,52]. Therefore it will be interesting to revisit this idea in clinical studies by directly measuring viral replication in lymphoid tissues - sites where cell-to-cell spread likely predominates and where compartmentalized viral replication may be supported. Under these conditions, one may speculate that PI-containing cART regimens may be more likely to have an impact on viral reservoirs.

## Conclusions

In conclusion we show that PIs are equally effective against both modes of HIV-1 spread between T lymphocytes and are more effective than RTIs in blocking equivalent cell-associated viral dissemination *in vitro*. Our data with RTIs support the previous suggestion that the variable effects of antiviral drugs on cell-to-cell spread of HIV-1 may impact ongoing viral replication, with the caveat that this would be dependent on both the drug-class and the multiplicity of infection (i.e. the number of infected cells relative to the *in vivo* drug concentration). Whether the high multiplicity of infection required to overcome RTIs occurs *in vivo* is difficult to evaluate. However, saturation of antiviral drugs by cell-to-cell spread may be more feasible in sanctuary sites such as lymphoid tissues, where cell-to-cell spread predominates and in which diffusion of antiviral agents may be reduced [1]. Therefore the variable effects of ART on cell-to-cell spread may need to be considered in future therapeutic strategies.

## Methods

### Cells, viruses and inhibitors

HeLa-TZMbl cells were obtained from the Center for AIDS Reagents, National Institutes of Biological Standard and Control, UK (CFAR, NIBSC) and donated by J. Kappes, X. Wu and Tranzyme Inc. HEK 293T cells were originally from the ATCC (American Type Culture Collection). Adherent cells were cultured in Dulbecco’s Modified Eagle Medium (DMEM) supplemented with 10% fetal calf serum (FCS, Invitrogen), 50 U of penicillin/ml and 50 μg/ml of streptomycin. The CD4^+^/CXCR4^+^ T cell line Jurkat CE6.1 and derivative Jurkat line 1G5 (obtained through AIDS Research and Reference Reagent Program, Division of AIDS, NIAID, NIH [ARRP]: from Dr. Estuardo Aguilar-Cordova and Dr. John Belmont) were maintained in RPMI 1640 supplemented with 10% FCS and 50 U/ml of penicillin and 50 μg/ml of streptomycin. The HIV-1 clone pNL4.3 was produced by Dr. Malcolm Martin and obtained from the ARRP. The HIV-1 PIs Lopinavir (LPV) and Darunavir (DRV) and the RTIs Nevirapine (NVP), Zidovudine (AZT) and Tenofovir (TDF) were obtained from the ARRP.

### Quantitative real-time PCR assay for cell-to-cell spread and inhibitor assays

Jurkat cells were infected by spinoculating at 1200× g for 2 h at an MOI of 0.1-0.3. HIV infected cells were used 48–72 hours post-spinoculation when >90% of the cells were infected. To measure cell-to-cell transfer a quantitative real-time PCR (qPCR) [32,33] with minor modifications. Briefly, HIV-1 infected cells were washed and 2×10^5^ cells per well were pre-incubated with protease inhibitors LPV or DRV for 24 h at 37°C. 8×10^5^ uninfected Jurkat 1G5 target cells were subsequently added and co-cultures were supplemented with fresh drug and incubated for 0 h, 1 h, 3 h, 6 h, 12 h or 24 h at 37°C after which the cells were pelleted, stored at −80°C and genomic DNA was extracted (QIAGEN). Quantitative real-time PCR was performed to measure cell-to-cell spread as described previously using primers and probes specific for HIV-1 *pol* DNA and the housekeeping gene *albumin*[32,33]. For experiments to calculate the IC50 of inhibitors, co-cultures were performed in the presence of a serial dilution of the inhibitor under study and incubated for 24 h before DNA extraction and qPCR. For cell-free infection experiments, virus produced from 2×10^5^ donor cells pre-incubated with drug for 24 h was used to infect 1×10^6^ target cells by spinoculating at 1200 *g* for 2 h. After 24 h incubation, the cells were pelleted, supernatant aspirated and frozen for DNA extraction and subsequently analyzed by qPCR*.* For RTIs, experiments were performed exactly as described above, except that uninfected target cells were incubated with Nevirapine (NVP), Zidovudine (AZT) or Tenofovir (TDF) for 1h at 37°C prior to the addition of HIV-1 infected donor cells. Fifty percent inhibitory concentrations (IC50s) were determined using Prism GraphPad Software.

For primary cell experiments, peripheral blood mononuclear cells (PBMC) were isolated from buffy coats (from the National Blood Transfusion Service, London, United Kingdom) by Ficoll-Hypaque (Sigma-Aldrich) gradient centrifugation. CD4+ T cells were isolated by negative selection according to manufacturer’s instructions (Miltenyi Biotec) and were routinely >90% pure. Primary CD4^+^ T cells were activated with RPMI supplemented with 1 μg/ml of PHA-L (Sigma) and 10 IU/ml of interleukin-2 (IL-2; NIBSC) for 3 days. CD4+ T cells were subsequently infected with HIV-1 at an MOI of 1 by spinoculating as described above. Three days later the cells were stained for Gag and analyzed by FACS to determine the % infection. HIV-1 infected primary CD4+ T cells were treated with LPV (C_max_ 14 μM), co-cultured with uninfected Jurkat 1G5 cells (targets) and cell-to-cell spread was quantified by qPCR as described above.

### Real-time PCR for the detection of 2-LTR circles

To quantify 2-LTR circles in the presence or absence of inhibitors, co-cultures of donor cells mixed with target cells with or without inhibitors were performed as described above. The cells were pelleted at 0 h and 24 h and total DNA extracted. 2-LTR circles were quantified in triplicate by qPCR as described by Apolonia *et al.*[53]*.* The primers used were 5′-AACT AGAGATCCCTCAGACCCTTTT-3′ and 5′-CTTGTCTTCGTTGGGAGTGAATT-3′, and Taqman probe 5′-CTAGAGATTTTCCACACTGAC-3′ [53]. To estimate the number of 2-LTR circles in target cells at the 24 h time-point all the values were normalized to the 0 h time-point which represents the number of 2-LTR circles in the donor cells at the time of mixing donors with targets.

### Flow cytometry

HIV-1 infected Jurkat cells were washed and fixed with 3% paraformaldehyde (PFA), permeabilized in BD™ Perm Buffer (BD Biosciences) and stained with anti-HIV p24 monoclonal antibody conjugated to fluorescein isothiocyanate (HIV-1 p24 (24–4) FITC, Santa Cruz Biotechnology) to detect intracellular Gag. Acquisition was performed using a Becton Dickinson FACS Calibur and data analyzed using FlowJo® software. Cells were used when >90% were Gag positive.

### Western blotting

Viral supernatants were harvested from HIV-1 infected T cells and virions purified by sucrose gradient centrifugation. Equal volumes of purified virus were separated by SDS-PAGE and analyzed by Western blotting using rabbit antisera raised against HIV-1 Gag that recognizes p55Gag and p24CA (donated by Dr G. Reid and obtained from the CFAR). Primary antibody was detected with goat anti-rabbit HRP (DAKO) and visualized by ECL (Amersham).

### Construction of a protease inhibitor resistance virus

To construct the drug resistant variant of NL43, site-directed mutagenesis was performed with Accuprime *Pfx* supermix (Invitrogen®) using forward and reverse primers containing the required nucleotide substitutions. Two mutations, V82A (mutagenesis primers: forward-5′- GTAGGACCTACACCTGCCAACATAATTGGAAG-3′, reverse-5′-CAGATTTCTTCCAATTATGTTGGCAGGTGTAGG-3′), a major protease drug resistance mutation and A431V (mutagenesis primers: forward-5′-GAAAGATTGTACTGAGAGAGACAGGTTAATTTTTTAGG-3′), a cleavage site-mutation in the p7/p1 junction, were introduced to make HIV-1_DM_ (DM = double mutant). The mutagenesis was carried out in the vector pCR® 2.1 TOPO, by sub-cloning a region of HIV-1_NL4.3_ covering nucleotides 740–2940. Sequencing was performed by BigDye terminator chemistry and a 3730xl analyzer (ABI®) to confirm the presence of the mutations introduced and the absence of any other substitutions. The mutated fragment was re-introduced into the HIV-1_NL4.3_ backbone by SpeI/AgeI digestion. Stocks of infectious virus (HIV-1_DM_ and HIV-1_WT_) were made by transfecting 293T cells using Fugene 6 (Promega). Infectious viral titers were measured on HeLa-TZMbl reporter cells using the Bright-Glo Luciferase assay kit (Promega).

### Drug susceptibility assay

An in-house assay was used to determine the drug susceptibility of the mutant virus compared to the wild-type vector [45]. The assay was modified to accommodate the use of plasmids with full-length HIV-1 genomes. HEK 293T cells were transfected as described above, 16 h later the cells were seeded in the presence of a serial dilution of PIs. Virus supernatant was harvested 24 h later and used to infect fresh target HeLa-TZMbl cells by spinoculating for 2 h at 1200× *g*. Replication was determined by measuring luciferase expression in infected target cells at 48 h post-infection using SteadyGlo luciferase assay system® (Promega) and expressed relative to that of no drug controls. Fifty percent inhibitory concentrations (IC50s) were determined using Prism GraphPad Software. The IC50 values calculated are the mean of at least two independent experiments.

### Statistical analysis

A two-tailed student *t*-test was performed to compare the mean IC50s for cell-free and cell-to-cell spread for PIs and RTIs. For comparisons of data with more than two groups a two-way ANOVA with Bonferroni post-test for multiple comparisons was used.

### Ethical approval

Experiments performed with blood from human volunteers was approved by the University College London Research Ethics Committee (ethical approval number 2649/001) in compliance with the Helsinki Declaration.

## Competing interests

The authors declare that they have no competing interests.

## Authors’ contributions

BT carried out the studies, participated in their design, analyzed the data and drafted the manuscript. MAC helped design experiments and analyze the data. DP conceived the study, participated in its design and helped draft the manuscript. CJ conceived the study, participated in its design, helped analyze data and helped draft the manuscript. All authors read and approved the final manuscript.
